# Assessment Methods of Post-stroke Gait: A Scoping Review of Technology-Driven Approaches to Gait Characterization and Analysis

**DOI:** 10.3389/fneur.2021.650024

**Published:** 2021-06-08

**Authors:** Dhanya Menoth Mohan, Ahsan Habib Khandoker, Sabahat Asim Wasti, Sarah Ismail Ibrahim Ismail Alali, Herbert F. Jelinek, Kinda Khalaf

**Affiliations:** ^1^Department of Biomedical Engineering, Health Engineering Innovation Center (HEIC), Khalifa University of Science and Technology, Abu Dhabi, United Arab Emirates; ^2^Neurological Institute, Cleveland Clinic Abu Dhabi, Abu Dhabi, United Arab Emirates

**Keywords:** post-stroke, gait, hemiplegia, machine learning, statistical tools, spatiotemporal, dynamics, artificial intelligence

## Abstract

**Background:** Gait dysfunction or impairment is considered one of the most common and devastating physiological consequences of stroke, and achieving optimal gait is a key goal for stroke victims with gait disability along with their clinical teams. Many researchers have explored post stroke gait, including assessment tools and techniques, key gait parameters and significance on functional recovery, as well as data mining, modeling and analyses methods.

**Research Question:** This study aimed to review and summarize research efforts applicable to quantification and analyses of post-stroke gait with focus on recent technology-driven gait characterization and analysis approaches, including the integration of smart low cost wearables and Artificial Intelligence (AI), as well as feasibility and potential value in clinical settings.

**Methods:** A comprehensive literature search was conducted within Google Scholar, PubMed, and ScienceDirect using a set of keywords, including lower extremity, walking, post-stroke, and kinematics. Original articles that met the selection criteria were included.

**Results and Significance:** This scoping review aimed to shed light on tools and technologies employed in post stroke gait assessment toward bridging the existing gap between the research and clinical communities. Conventional qualitative gait analysis, typically used in clinics is mainly based on observational gait and is hence subjective and largely impacted by the observer's experience. Quantitative gait analysis, however, provides measured parameters, with good accuracy and repeatability for the diagnosis and comparative assessment throughout rehabilitation. Rapidly emerging smart wearable technology and AI, including Machine Learning, Support Vector Machine, and Neural Network approaches, are increasingly commanding greater attention in gait research. Although their use in clinical settings are not yet well leveraged, these tools promise a paradigm shift in stroke gait quantification, as they provide means for acquiring, storing and analyzing multifactorial complex gait data, while capturing its non-linear dynamic variability and offering the invaluable benefits of predictive analytics.

## 1. Introduction

Stroke, defined as the sudden rupture or blockage of a cerebral blood vessel and consequent damage to central nervous system cells and tissues due to the interruption of oxygen supply, remains a major health challenge throughout the world. According to the World Health Organization (WHO), every year, 15 million people worldwide are diagnosed with stroke, of which, approximately 6 million die and another 5 million are left with permanent disabilities (1). Indeed, stroke is globally considered as the second leading cause of mortality for individuals above the age of 60 years, and the fifth leading cause of death in individuals aged 15–59 years (2).

While developed countries are reporting an overall decline in the incidence of stroke in the population below 65 years of age, its incidence is increasing in the developing world. Predictions for the next two decades indicate tripling in stroke mortality in Latin America, the Middle East, and Sub-Saharan Africa. In the United Arab Emirates (UAE), stroke is the second leading cause of disability, next to road accidents. Based on statistics from the Department of Health in Abu Dhabi (DOH), approximately 8,000–10,000 Emiratis experience a stroke each year, translating to at least one stroke occurring every hour (3). In addition to the high prevalence, 50% of stroke patients in the UAE are below the age of 45 years, which is approximately 20 years younger than the global average (3). Despite increased awareness and lifestyle changes, post stroke rehabilitation based on accurate and repeatable, objective assessment that leads to individualized rehabilitation protocols, and improved long-term mobility and quality of life is yet to be implemented on a large scale.

A variety of standardized stroke scales are currently used by clinicians to quantify stroke impairments, including neurological deficits and gait abnormalities (4) (see Table 1). Gait abnormalities in stroke patients are predominantly due to sensorimotor dysfunction, including muscle weakness, perceptual and proprioceptive deficits, spasticity or hypotonia. These impairments can affect a stroke victim in various degrees and combinations depending on the severity of the stroke (level A or minimal neurological deficit; level B or moderate deficit; and level C or severe deficit). Walking dysfunction for individuals with post-stroke gait impairment is often characterized by abnormal kinematic and kinetic patterns, deviations in the spatiotemporal features, altered muscle activation, and increased energy expenditure during walking. Early and effective rehabilitation with appropriate pharmacological and therapy interventions can help regain good ambulatory function with optimized modified gait patterns. While 52–85% of hemiplegic stroke patients regain their walking capacity, their gait patterns typically continue to differ from those of healthy individuals, with negative impact on biomechanics, overall body function, and quality of life (17, 18). Reduced walking speed, shorter and narrower steps, the inability to walk a mile (1,609 m) or difficulty to ascend a flight of stairs are observed to contribute to post-stroke disability (19).

**Table 1:** Stroke scales and characteristics.

**Tool**	**Year**	**No. of test items/ components**	**Time to administer**	**Tool format**	**Score summary**	**Description**
Barthel Index (5, 6)	1955	10	5 min	Each task uses different scores from (0, 5, 10, 15)	0–100; least to great independence	Measurement of functional independence in stroke patients
Modified Rankin Scale (5)	1957	6 items	5 min	6-Point ordinal scale (0–5); score of 6 added denote death	0–5; no symptoms to severe disability	Describes the degree of disability in daily activities of people with stroke or other neurological disorder
Hunt & Hess Scale (7)	1968	5	NA	Not weighted	1–5; minimum to maximum mortality	Prediction of prognosis and outcome in patients with subarachnoid hemorrhage
Mathew Stroke Scale (8, chap.9)	1972	10	15 min	Arbitrarily weighted	100 point scale; lower scores reflect a more severe deficit	Measurement of stroke severity in clinical trials; designed for study on glycerol therapy
Glasgow Coma Scale (GCS) (9)	1974	3 components	2 min	Tasks graded using 4 (1–4), 5 (1–5), and 6 (1–6) point ordinal scale	3–15; Deep comma to fully awake	Assessment of level of consciousness (LOC) for acute medical and trauma patients
Glasgow Outcome Scale (GOS) (8, chap.9)	1975	5 items	Few seconds	Not weighted	1–5; dead to a good recovery	Used for categorizing the outcomes of patients after traumatic brain injury
Fugl-Meyer assessment scale (10)	1975	28	35 min	Ordinal scale	172 point scale	Used to assess motor and joint functioning, balance, and sensation in stroke patients with hemiplegia
Toronto stroke scale (10)	1976	11 categories	NA	NA	0 to 155	Used for evaluating acute stroke patients
Orgogozo Stroke Scale (8, chap.9)	1983	10	10 min	Ordinal scale	0–100; severe to normal	Used for patients with middle cerebral artery infarction
Functional Independence Measurement (FIM) (8, chap.9) (6)	1984	18	30-45 min	7-Point ordinal scale, 1 (requiring complete dependence) to 7 (completely independent)	18–126; complete dependence to complete independence	Used for assessing a patient's level of disability
Canadian Neurological Stroke Scale (CNS) (5, 10, 11)	1986	8	5–10 min	Each section uses different scores from (0,0.5,1,1.5, 3)	1.5–11.5; lower to greater neurological deficit	Evaluation and monitoring of acute-stroke neurological status
Hemispheric Stroke Scale (8, chap.9) (10, 12)	1987	20	15-30 min	Ordinal scale	0–100; Good to bad	Assessment of neurological deficit in stroke therapy using hemodilution
Modified Mathew Stroke Scale	1988	10	NA	Ordinal scale	NA	Used in nimodipine and hemodilution studies for acute stroke
Copenhagen stroke scale (13, 14)	1988	10 item	<10 min	Ordinal scale; *a* (normal) to *f* (worse) and *a* to *d* in the revised one	NA	For estimating the initial severity of stroke
NIH Stroke Scale (NIHSS) (15)	1989	15	7 min	Each scores between 0 and 4	0–42; No stroke symptoms to severe stroke	Measurement of neurological deficit in acute stroke patients
Scandinavian Stroke Scale (6, 10, 13)	1992	9	5 min	Ordinal scale	0–58; very severe to mild	Designed for non-neurologists for multicenter hemodilution trials
European Stroke Scale (16)	1994	14	8 min	Arbitrarily weighted tasks	0–100; maximally affected to normal	Detection of therapeutic effect and matching of treatment groups for middle cerebral artery stroke
Japan stroke scale (15)	NA	10	NA	Weighted tasks	NA	Measuring stroke severity

*NA, not available*.

### 1.1. Major Determinants of Ambulatory Function/Mobility in Stroke

Gait velocity of individuals with post-stroke gait impairment ranges from approximately 0.18 to 1.03 m/s, whereas that of healthy age-matched adults has an average of 1.4 m/s (20). Identifying the clinical features that are primarily associated with post-stroke walking ability is critical for the development of effective gait-training programs. In particular, impairments in muscle strength, motor function, and balance have been observed to be highly correlated with walking ability. The muscle strength of the affected hip flexor, ankle plantar flexors, knee extensors, knee flexor, as well as that of unaffected knee flexors and ankle plantarflexors are moderate to highly correlated (*r* = 0.5~0.8) with walking and stair climbing speed (20, 21). Motor function of the affected lower limb as rated by Fugl-Meyer Assessment is significantly correlated with gait velocity of patients with mild to moderate stroke (*r* ~ 0.6) (22). Indeed, balance function, as measured on the Berg Balance Scale, was highly correlated with functional walk distances [6 Min Walk Test (6 MWT) and 12 Min Walk Test (12 MWT)] at self-paced speed (*r* = 0.78–0.80) (23).

Other stroke-induced impairment, including spasticity, sensory function, and cardiovascular fitness have less impact on walking, and the association between these impairments with the walking velocity remains controversial (20). The correlation between the degree of spasticity and walking speed was also found non-significant (20, 24). Keenan et al. reported that tactile and proprioception impairments affect walking ability (25). However, Nadeau et al. and Dettmann et al. found no significant correlation between lower limb sensation and gait speed (22, 26). Further, cardiovascular fitness [Peak oxygen uptake (Vo2 peak)] was observed to be moderately associated with 6 MWT distance (*r* = 0.56) in sub-acute stroke (27).

### 1.2. Post-stroke Gait Assessment

In clinical gait assessment, both a person's “ability” to walk and “how” the individual walks are relevant. Walking ability of a person with stroke is a function of the stroke severity and is typically based on two main aspects: how far can an individual walk and what is his/her tolerance level. These are usually assessed using 3-, 6-, or 10- min walk tests. Functional Ambulation Category (FAC), Short Physical Performance Battery (SPPB), and/or Motor Assessment Scale (MAS) may also be employed for further assessment. On the other hand, the quality of gait or “how” the person walks is based on studying gait patterns and specific gait characteristics. Studied much more often in research settings rather than clinical settings, gait characteristics are nowadays assessed using instrumented gait analysis, including kinematic and kinetic assessment, which are beneficial for clinicians toward setting patient-specific quantitative functional ambulatory benchmarks and goals.

During the early stages of post-stroke recovery, patients often undergo qualitative observational, also referred to as visual gait analysis (using naked eye or video images) by physicians to evaluate gait performance and functional improvement (28). Table 2 lists various observational gait scales widely practiced in clinical settings. Current clinical assessment methods based on visual observation rely heavily on training and clinical judgment. However, despite being scrutinized for inter-observer variability (36), “observational gait analysis” methods continue to be popular among clinicians. This is due to the simplicity and availability of these tools, as well as their low cost (37). Nevertheless, validity, reliability, specificity, and responsiveness (38, 39) of these qualitative methods are questioned (36). In general, subjective observational gait scales may be useful for the rudimentary evaluation of some spatiotemporal and/or kinematic gait parameters but are not adequate for analyzing the multifaceted aspects of gait variability and complexity (for example kinetic and balance parameters). There is no consensus currently to when a more sophisticated gait analysis should be undertaken in stroke patients. The timing is likely to depend on the severity of the stroke, as well as parameters including fatigue, instability, pain, and consistent poor walking patterns despite fair to good muscle activity during passive examination.

**Table 2:** Observational gait scales and characteristics.

**Tool**	**Gait parameter**	**No. of test items**	**Tool format**	**Time to administer**	**Score summary**
Gait Assessment and Intervention Tool (G.A.I.T) (28)	kinematics	31	2 to 4-level ordinal scale	20 min, not including videotaping	0–62; normal to greatest extent of gait deviation
New York Medical School Orthotic Gait Analysis, (NYMSOGA) (29)	kinematics, spatiotemporal	17	3-level ordinal	not reported	not reported
Hemiplegic Gait Analysis Form (HGAF) (30)	kinematics, spatiotemporal	18	3-level ordinal scale	not reported	0–88; normal to abnormal gait
Rivermead Visual Gait Assessment (RVGA) (31)	kinematic	20	4-level ordinal scale	10–15 min	0–59; normal to abnormal gait
Wisconsin Gait Scale (WGS) (32, 33)	kinematics, spatiotemporal	14	3, 4, and 5-level ordinal scale	35–45 min for video recording and offline processing	13.35–42; normal to worst
Tinetti Gait Scale (TGS) (34)	kinematic	8	2 and 3-level ordinal scale	5 min	0–12; most deviation to normal
Gait Abnormality Rating Scale - modified (GARS-M) (35)	kinematics, spatiotemporal	7	4-level ordinal scale	not reported	0–21; low to high risk of falling

Instrumented gait analysis, which has become standard in research settings in the 1990's, provides an accurate, reliable biomechanical gait evaluation approach incorporating key parameters (spatiotemporal, kinematic, and kinetic measures) (40). Gait labs typically include large, cumbersome, and expensive equipment, such as motion capture systems (Vicon, Motion Analysis Inc., Qualisys, OptiTrack, etc.), force plates (Bertec, Kistler, Noraxon, etc.), sensor-embedded walkways (Tekscan, GaitRite, etc.), and balance platforms, in addition to any other instruments deemed important for particular research applications, including post stroke gait assessment. Construction and configuration of these labs are specific to allow for accurate testing. For example, an area of at least 9 meter wide by 11 meter long with 3–5 meter ceilings (higher ceilings for stair climbing and sports applications) and special floors. Not surprisingly, the implementation of these labs in clinical settings remains scarce, not only due to the construction challenges and cost, but also the limited number of clinical practitioners appropriately skilled to conduct the tests and manage/interpret the large amount of generated data obtained from the array of possible sensor technology in use in post stroke gait rehabilitation. On the other hand, the clinical efficacy of 3-dimensional instrumented gait analysis (3DGA) has improved this last decade as reported by Wren et al. in their systematic reviews conducted in 2011 as well as in 2020 (41, 42). They classified studies based on the type of efficacy the studies addressed, which included technical, diagnostic, outcome prediction, diagnostic thinking and treatment, patient outcome, and societal efficacy. There is strong evidence suggesting a continued advancement of technology, including system accuracy and reliability, and data collection and analysis. From the clinical perspective, there is an increased use of 3DGA for understanding and evaluating the efficacy of treatment at a group-level. Also, the impact of 3DGA in treatment decision-making is evidenced. This includes diagnosing gait deficit and associated causes (e.g., toeing), devising treatment options (e.g., surgical or non-surgical), as well as changing and reinforcing treatment plans. In addition, the efficacy on individual treatment outcomes was also supported by studies. A scarcity of research analyzing the cost-effectiveness of 3DGA at a societal level recommends the need for further research toward this aspect (41, 42).

Today, small, light wearable sensors, such as inertial measurement units (IMU), pressure sensors, accelerometers, and various types of smart wearables are rapidly revolutionizing gait assessment in research settings and have the potential to be included in routine clinical practice. These sensors offer new opportunities for researchers to continuously record gait allowing the application of methods that quantify gait dynamics over time and can provide real-time feedback to patients and clinicians. Their light weight, portability and low-cost offer potential for research outside the lab and in natural environments (clinics, sports arenas, etc.). In addition, these sensors allow for easy synchronization with other physiological measurement equipment, such as EMG, ECG, and EEG, providing invaluable multifactorial continuous data of the subject/patient in various settings.

Recently, artificial intelligence, including machine learning techniques has emerged as a promising tool for processing instrumented gait data efficiently (43–47). These techniques have found applications in numerous problems relating to gait assessment, including dimensionality reduction, feature extraction, and classification. For instance, Zhou et al. employed Support Vector Machine (SVM), Random Forest (RF), and Artificial Neural Network (ANN) to classify young middle-aged, older adults, and geriatric patients, based on dynamic gait outcomes (46). Kernel Principal Component Analysis (KPCA) was incorporated for dimensionality reduction of the data for SVM classifiers. Lau et al. implemented SVM, ANN, and Radial Basis Function neural networks (RBF) classifiers to classify different walking conditions of hemiparetic patients, and found that SVM resulted in a highest overall classification accuracy of 97.5% (44). In addition, Lee et al. applied general regression NN for decision making process (43), whereas, Scheffer and Cloete used ANN, which was optimized to distinguish between hemiparetic stroke and able-bodied ambulation (45). These AI-based techniques have demonstrated capability of systematically analyzing and extracting information from an extensive amount of instrumented gait data that are multi-dimensional, highly non-linear, and/or too complex for analysis using traditional statistical approaches.

Considering the relevance of post stroke gait analysis in a clinical context and the potential value of a technological paradigm shift in that domain, this study includes the review of current literature up to January 2021 on post-stroke gait analysis with focus on conventional and advanced gait analysis tools and techniques that are low cost, commercially viable, easy to use, transportable and do not require excessive training for potential use in clinical practice. In particular, this review highlights:

1. basic measurement protocols required to assess post-stroke gait in clinical and/or research settings based on findings from the literature.
2. data processing and analysis protocols used by various researchers and the functional applicability of different techniques.
3. characteristics of gait in individuals with post-stroke gait impairment and the significant parameters used to assess gait deficits following a stroke.

This scoping review is primarily dedicated to bridging the gap between recent technology-driven engineering research gait studies and clinical applications, particularly in the area of post stroke gait. This gap can be seen in the faster adoption of the rapidly emerging gait assessment/analysis tools and technology in research labs as compared to clinical settings. Nowadays, gait studies can be performed using cost-effective, reliable and wearable sensors. These are invaluable when integrated into functional clinical application, where real-time gait analysis could be key to the development of patient-specific gait rehabilitation strategies and techniques. The main goal of this review is to provide a practical resource on technology-driven gait characterization and analyses tools and techniques and discuss their value and feasibility for clinical practice. The remainder of the review is structured as follows: section 2 describes the adopted methodology, including the approach, search strategy and selection criteria. Section 3 highlights the attributes of normal and pathophysiological gait, including post-stroke gait and mobility. Conventional and technology-driven gait characterization tools and technologies are reviewed in section 4, while section 5 summarizes associated conventional and technology-driven data processing and gait analysis. Section 6 sheds light on relevant gait and physiological parameters used to characterize post-stroke gait both in research labs and clinical settings. Novel state-of-the-Art AI techniques for gait analysis are described in section 7. Section 8 details the limitations of the study, while section 9 presents the conclusions and future work.

## 2. Methods

### 2.1. Study Approach

This paper appraises or evaluates the current use of biomechanics and physiological data recording and analyses for the assessment of post-stroke gait, as well as potential future directions including smart wearables and big data/artificial intelligence-based techniques. One of the main goals for this review is to serve as a practical resource for both engineers and physicians who deal with post-stroke gait assessment in research labs as well as clinics. As such, a broad topic area, including gait and its multifactorial physiological parameters, measurement tools and protocols, data analysis techniques, and future trends including AI and ML is included in this review.

As highlighted in (48), scoping reviews are an ideal tool for determining the coverage of a body of literature pertaining to the topic under study, determining the volume of the available studies, as well as, providing a broad or detailed overview of the focus. It can be used as a precursor to a systematic review. Scoping reviews also generally address a broader research question as compared to systematic reviews, and hence typically include more extensive inclusion criteria (48). Considering the nature of this study and intent, which are to identify the type of evidence and provide a broad overview about a wide scope of the aforementioned research area, the authors believe that a scoping review rather than a systematic review would best benefit both research and clinical communities.

### 2.2. Search Strategy

**Figure 1:**
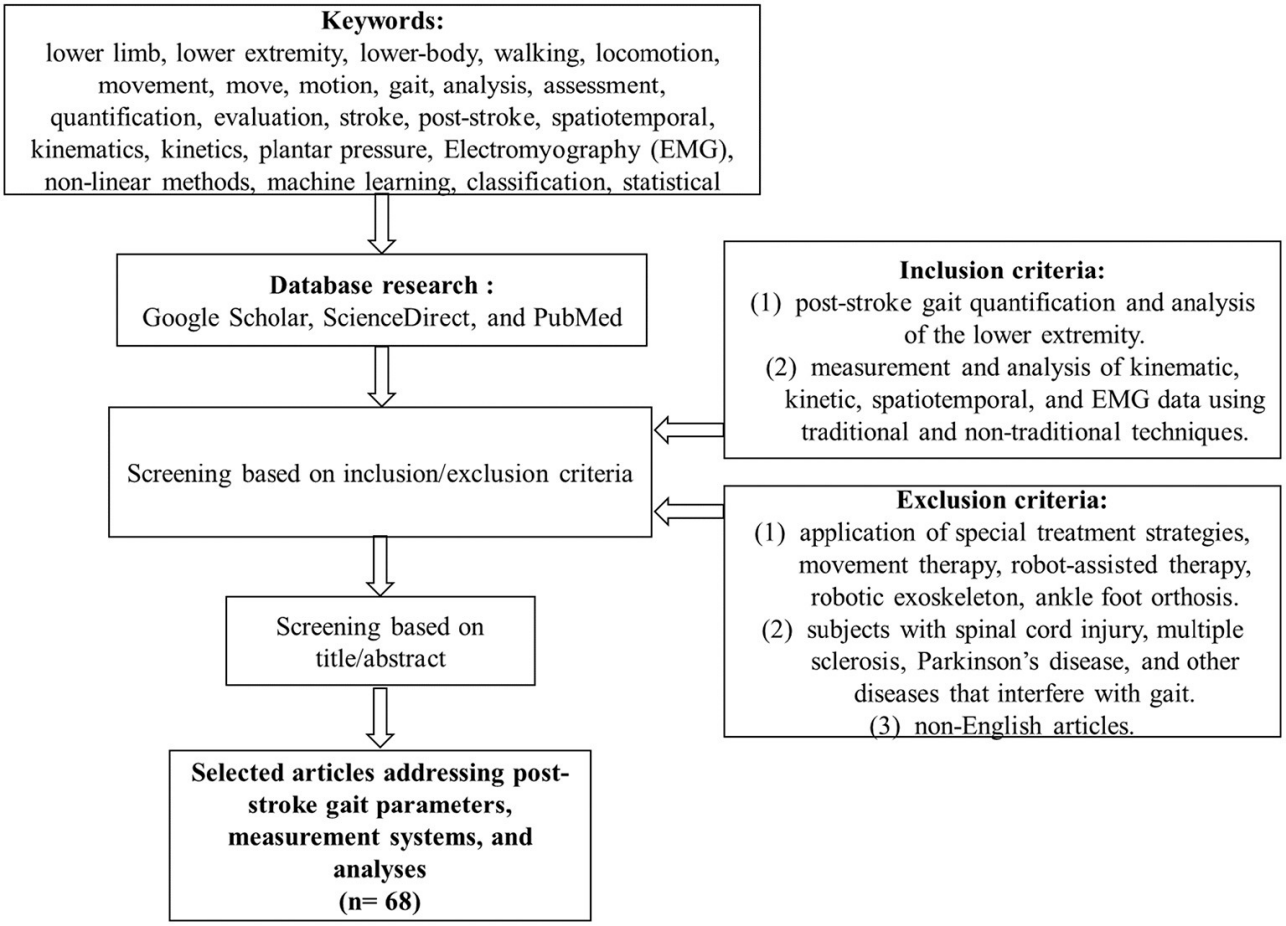
Flowchart of the search.

A comprehensive literature search was conducted within Google Scholar, ScienceDirect, and PubMed databases using a combination of keywords from the following groups: (1) “lower limb” or “lower extremity” or “lower-body”; (2) “walking” or “locomotion” or “movement” or “move” or “motion” or “gait”; (3) “analysis” or “assessment” or “quantification” or “evaluation”; (4) “stroke” or “post-stroke”; (5) “spatiotemporal” or “kinematics” or “kinetics” or “plantar pressure” or “Electromyography (EMG)” or “non-linear methods” or “machine learning” or “classification” or “statistical.” The search was limited to articles published in English until January 2021. The title and abstract of the search results were screened against the inclusion/exclusion criteria. The full text of the relevant articles was reviewed and those meeting the study criteria were considered for further analysis. The references were checked to identify additional articles for possible inclusion in this study (see Figure 1).

### 2.3. Study Selection Criteria

Articles were screened against the following inclusion/exclusion criteria. Articles for inclusion had to address (1) characteristics of post-stroke gait including biomechanical and physiological parameters, or (2) post-stroke gait quantification and analysis of lower extremity, or (3) gait measurement system/protocol, or (4) data analysis using traditional and non-traditional techniques. Studies addressing a sample population of post-stroke patients, who are able to walk are considered in this review. Studies that involve the application of special treatment strategies (e.g., use of botulinum toxin injection, aerobic exercise, etc.), movement therapy, robot-assisted therapy, and studies with robotic exoskeleton and/or ankle foot orthosis are outside the scope of this study and not included in the review. Articles analyzing subjects with spinal cord injury, multiple sclerosis, Parkinson's disease, and other diseases that interfere with gait were also excluded.

The articles meeting the inclusion and exclusion criteria were screened for value provided in gait quantification and analysis of post-stroke patients. The measurement methods were studied in detail, and the parameters considered were identified. The various techniques to analyze gait data and their applicability for clinical diagnostics/prognostics and personalized medicine were further examined.

### 2.4. Data Extraction

Following the screening of the full-text articles based on the criteria, a primary reviewer (DMM) performed the data extraction. A part of the data extraction was also carried out by an additional reviewer (SIIIA). The results were verified by (KK, HFJ, and AHK). The clinical application and relevance were discussed and confirmed by our clinical collaborator (SAW). The data extraction focused on three broad areas: characteristics of post-stroke gait, experimental/measurement/study protocol, and data analysis techniques. The characteristics included both biomechanical (e.g., spatiotemporal, kinematics, kinetics) and physiological parameters (e.g., heart rate variability). The measurement systems consisted of conventional techniques (e.g., footprint method) as well as the advanced techniques (e.g., pressure mat, motion capture, wearable sensors, etc.). The data analysis techniques incorporated statistical-based (e.g., ANOVA, linear regression, etc.) approaches as well as data-driven methods. Additionally, artificial intelligence techniques (e.g., ML, SVM) applicable to gait data analysis were also reviewed.

## 3. Introduction to Normal Gait and Pathophysiology of Human Gait

### 3.1. Normal Gait Pattern

**Figure 2:**
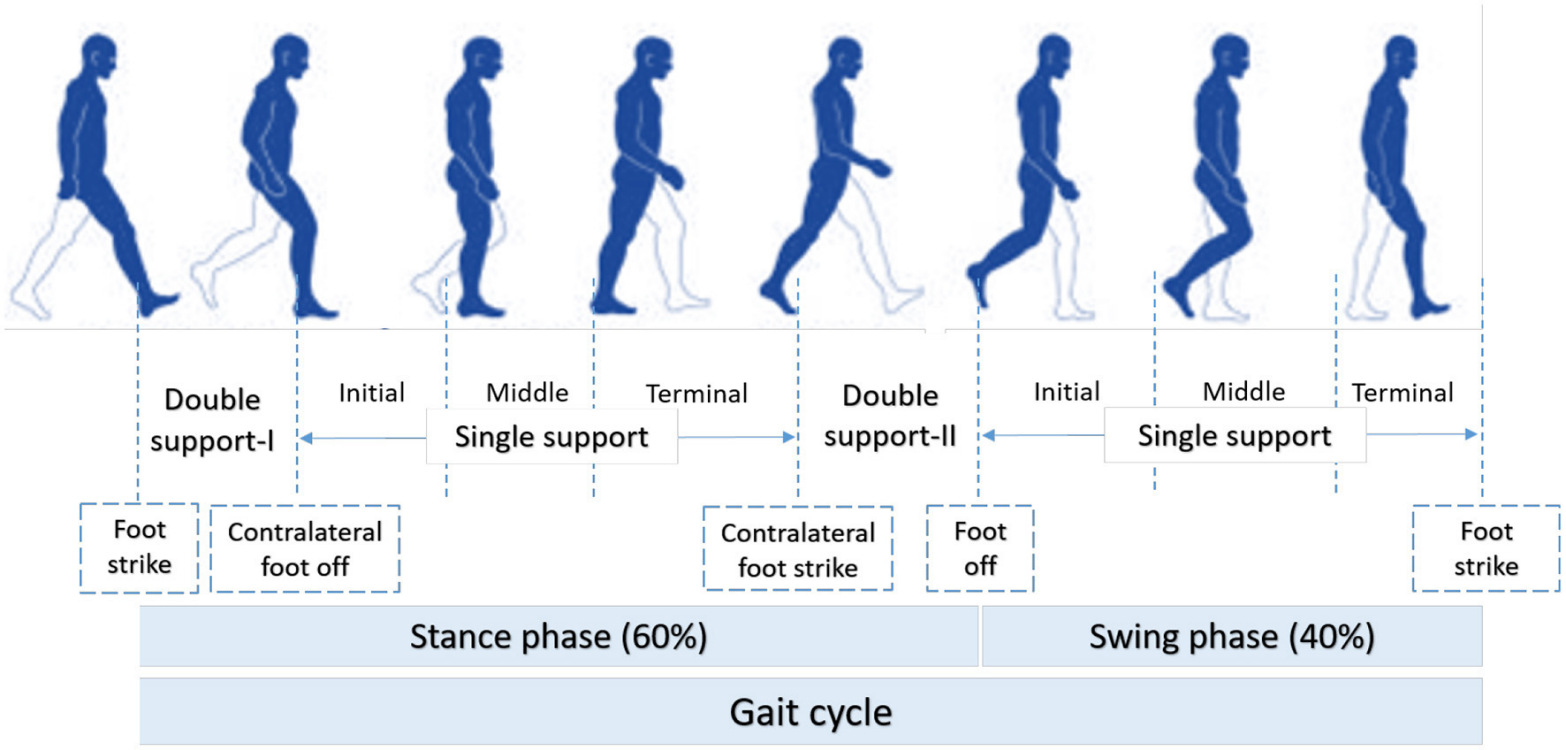
Functional phases of a normal gait cycle according to (50).

According to (49), normal gait can be defined as a series of rhythmic, systematic, and coordinated movements of the limbs and trunk that results in the forward advancement of the body's center of mass. As such, human movement is characterized using individual gait cycles and functional phases. As illustrated in Figure 2, a gait cycle consists of two main phases, stance and swing, which are further divided into five and three functional phases, respectively (51, 52). The stance phase corresponds to the duration between heel strike and toe-off of the same foot, and it constitutes approximately 60% of the gait cycle. The swing phase begins with toe-off and ends with heel contact of the same foot and occupies 40% of the cycle. Each functional phase contributes to successfully establishing the goal of walking. Any abnormalities observed in the phases or events of gait may be linked to musculoskeletal and/or neuro-muscular complications, such as the case of individuals with post-stroke gait impairment.

Many studies (53–62) have been conducted on healthy gait in order to derive a quantitative baseline for normal gait in terms of parameter ranges, so that deviations from the baseline could be identified as impairment or adaptation and proper treatment/rehabilitative therapies put in place. Adaptation may refer to any gait deviation that arises when an action is taken to mitigate the effects of an impairment. Table 3 provides gait parameter ranges as observed in studies on healthy adults. Determining an appropriate normal range for many of the features is highly challenging as individuals exhibit a wide range of gait patterns across different age groups and gender.

**Table 3:** Typical gait parameters of an adult healthy population.

**Parameters (self-selected speed)**	**Range**
Gait velocity (m/s)	1.30–1.46
Stride length (m)	1.68–1.72
Step length (m)	0.68–0.85
Stance phase (s)	0.62–0.70
Swing phase (s)	0.36–0.40
Cadence - fast walking (steps/min)	113–118
Single support (% of stride)	60.6–62.0
Double support (% of stride)	21.2–23.8

*Data adapted from (56). Ranges are indicative of subject populations and do not necessarily hold for the general population*.

### 3.2. Stroke and Pathophysiology of Human Gait

Stroke patients may exhibit deficits in muscle strength and muscle tone, mobility, perception and motor-control, sensation, and balance (63, 64). This leads to significant changes in voluntary movement, thereby affecting gait patterns. Gait deviations in post-stroke patients are divided into (i) primary deviations, defined as those directly due to pathology, and (ii) secondary deviations, which are the result of the physical effects of the primary deviations (passive), or a compensatory mechanism (active).

**Figure 3:**
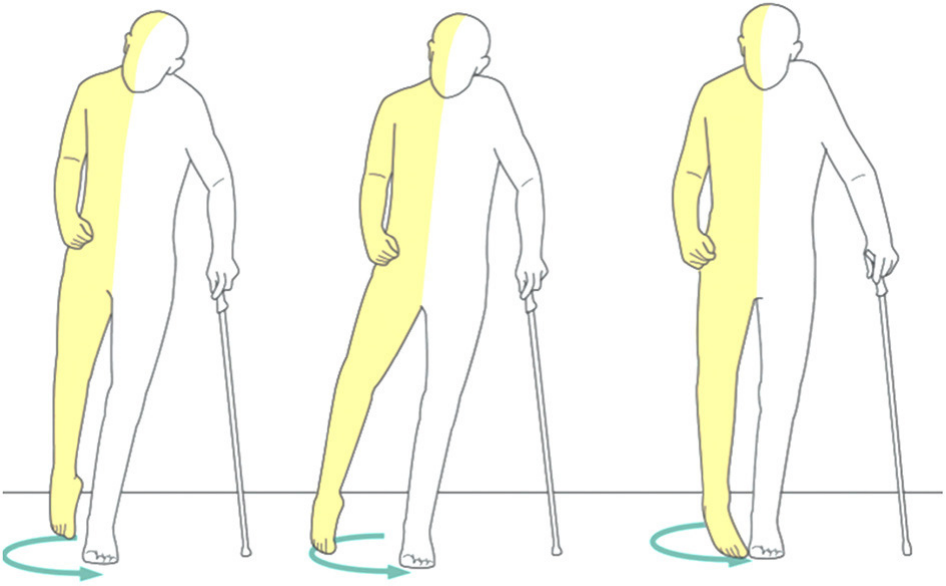
An example of uncorrected post-stroke spastic gait pattern (66).

Reduced gait speed and poor adaptation to daily-life tasks and environmental constraints are often observed (65) in persons with stroke. In sub-groups of stroke patients, functional adaptations include asymmetric steps, reduced weight bearing on the paretic limb, and reduced intra- and inter-limb coordination, leading to various compensatory adjustments including pelvic obliquity, hip hiking, and hip abduction with circumduction pattern to achieve foot clearance (66) (Figure 3). Kinematic deviations include reduced amplitude of the joint angle profiles at the lower extremities, whilst kinetic deviations include reduced joint moment and power. In addition, drop-foot is considered to be another major deficit following a stroke.

### 3.3. Post-stroke Mobility

Independent mobility after stroke is key to intervention during the various stages of post-stroke. Mobility is defined as “the ability to move oneself (e.g., by walking, by using assistive devices, or by using transportation) within community environments that expand from one's home, to the neighborhood, and to regions beyond” (67). The assessment of functional mobility after stroke is very important as it determines the effectiveness of rehabilitation strategies.

A Timed Up & Go (TUG) task is a simple tool employed to assess improvements in functional mobility over time. A TUG task starts with a patient in a seated position in a chair. It calculates the time taken for the subject to stand up, walk 3 meters at a comfortable speed, turn around, walk back to the chair, and sit down. According to Persson et al., based on a study involving 91 patients with first-ever stroke during their 1^st^ week, and at 3, 6, and 12 months post-stroke, the TUG time has reduced from 17 to 12 s between the 1^st^ week and 3 months, with no statistically significant changes afterwards (68). Buvarp et al. investigated the longitudinal progression as well as the change in functional mobility between moderate and mild stroke (69). They reported improvement in functional mobility of moderate stroke group at 1 year post-stroke [patients with age <75 improved by 7.2 s in TUG time (*P* < 0.001), and patients with age ≥75 years improved by 5.8 s (*P* = 0.011)]. For the mild stroke group, no statistically significant improvement was reported.

Other methods that assess walking and balance of persons with stroke at the level of community walking include Step Test, Side-Step Test, and Four-Square Step Test that assess a single task, and Brunel Balance Assessment, Dynamic Gait Index, Modified Emory Functional Ambulation Profile, Community Balance and Mobility Scale, and mini-Balance Evaluation Systems Test that assess multiple tasks (70). Based on clinical utility, single-task measures may be recommended to use as screening tools or to identify basic components of walking and balance. On the other hand, multi-task measures enable a comprehensive evaluation of walking and balance and could be used for identifying mobility deficits and devising treatment strategies. Readers are directed to (70, 71) for a summary of the psychometric properties of these assessment techniques.

## 4. Tools and Techniques for post-Stroke Gait Characterization

Gait analysis typically involves measurement, quantification, assessment, and interpretation of parameters that characterize bipedal walking or gait. Measurement covers a range of techniques available for recording gait events. Quantification phase includes the extraction of the various biomechanical parameters of gait (spatiotemporal, kinematic, kinetic), while assessment is concerned with the application of analytical and computational techniques, and interpretation consists of inferring the implicit factors affecting the walking mechanism.

Considerable research efforts have been devoted to the measurement of gait parameters ranging from simple stop watches and measuring tapes, to emerging sophisticated hardware and software technologies including lightweight wearable sensors. This section will review both traditional and novel techniques applied for measuring gait characteristics as identified in the literature (see Table 4).

**Table 4:** An overview of the literature focusing on gait parameters and measurement devices for post-stroke gait studies considered in this review.

**References**	**Gait parameters**	**Sample**	**Time post-stroke**	**Measurement/Protocol**	**Observation**
Moseley et al. (72)	Segmented kinematics	n/a	n/a	n/a	Decreased peak hip extension in the late stance phase; Decreased peak lateral pelvic displacement in stance phase; Increased peak lateral pelvic displacement in stance phase; Decreased knee flexion (or knee hyperextension) in stance phase; Increased knee flexion in stance phase; Decreased ankle plantarflexion at toe-off.
Moore et al. (73)	Segmented kinematics	n/a	n/a	n/a	Decreased peak hip flexion and ankle dorsiflexion in swing phase; Reduction in the peak knee flexion in early swing phase; Decreased knee extension prior to heel strike;
Nickel (74)	Gait velocity, gait cycle time, cadence, stride length, total double support time, single support time, duration of stance phase, duration of swing phase	49 stroke patients and 24 controls (controls had either transient ischemic episodes or asymptomatic carotid stenosis, symmetrical gait without walking support); time since stroke	avg 43.4 (range 0.5 to 336) months	Portable stride analyser, an insole system with compression foot switches (B& L Engineering, Santa Fe Springs, CA).	Cadence and velocity improved over time; Asymmetric patterns did not change over time; Age-matched controls in this study showed abnormal gait behavior compared to normal subjects.
Olney et al. (75)	Spatiotemporal, joint kinematics, moments, mechanical work and power	31 hemiplegic stroke patients	avg 11.4 (range 2.0 to 88.0) months	2D motion capture system (LoCam 51 camera); 3 trials;	Use of principal component analysis (PCA) for clustering of variables.
Silver et al. (76)	Walking speed, cadence, gait cycle symmetry (intralimb stance-swing ratio, interlimb stance duration ratio, interlimb swing ratio, overall stance-swing ratio)	5 post-ischemic stroke patients (mild to moderate gait asymmetries due to residual hemiparesis)	26 ± 4.6 (range 9 to 70) months	Videotape (Peak Motus Video Analysis system); modified Get-Up and Go task.	Improvements in walking speed and cadence, reduction in time required to complete the task; Sophisticated kinematics and kinetics analysis required to draw further results.
Woolley (77)	Distance and temporal parameters, joint kinematics, kinetics, mechanical power, energy expenditure, electromyography	n/a	n/a	n/a	Many gait deviations in the hemiplegic patients may be related to reduced walking velocity.
Hesse (78)	Stance and swing time symmetry, ground reaction forces, muscle activity profile, cardiovascular fitness	Hemiparetic subjects	n/a	10 meter test most commonly used; 2 walking trials	10-meter test and 6 min test are highly recommended to derive basic gait parameters; Abnormal muscle activity observed in stroke population; trajectory of vertical forces and center of pressure varies between controls and post-stroke patients; appearance of stance and swing time asymmetry.
Hsu et al. (20)	Gait velocity, step length asymmetry ratio, single support time asymmetry ratio	26 stroke patients (those with limited lower-body joint range of motion, joint pain, and history of unstable medical conditions, neurological, and/or musculoskeletal issues were excluded)	avg 10.3 (range 1 to 43) months	GaitMatII (EQ Inc., Plymouth Meeting, PA) (3.8 m); Cybex 6000 isokinetic dynamometer (Cybex International Inc., Medway, MA) to measure isokinetic muscle strength; 6 trials per speed condition; comfortable- and fast-speed	The weakness of the affected hip flexors and knee extensors contribute to a decrease in gait velocity; The spasticity of the affected ankle plantarflexors causes asymmetry.
Patterson et al. (79)	Stance time, swing time, double support time, intra-limb ratio of swing-stance time, step length, spatiotemporal symmetry	161 stroke patients and 81 age-matched healthy subjects	avg 23.7 (SD 32.1) months	GAITRite (10 m); 3 trials	Ratio equation can be used for standardization due to its clinical utility; Swing time, stance time, and step length are the most useful gait parameters
Patterson et al. (80)	Velocity, spatiotemporal symmetry	171 stroke patients data (first-ever unilateral stroke; hemorrhagic or ischemic)	avg 23.3 (SD 31.1) months	GAITRite mat (CIR Systems Inc., New Jersey, USA); 3 trials; preferred/comfortable speed	Swing time, stance time, and step length asymmetries may progress in the long term post-stroke stages; In terms of gait velocity and neurological and motor deficit, no difference is seen across the stages.
Laudanski (81)	joint angles of hip, knee, and ankle	10 chronic hemiparetic stroke patients and 10 healthy controls	6.5 ± 5.4 years	7 IMU sensors (Xsens Technology B.V., Netherlands), placed at midthigh, midshank, midfoot, and pelvis; Optotrak 3020 system (Northern Digital Inc., Ontario, Canada) for validation; force plates (AMTI, Newton, MA); 3 trials; self-selected speed	IMU-based systems are suitable for lower limb major joint angle estimation of healthy subjects and range of motion estimation of stroke patients. Additional calibration techniques are required for the application in stroke population.
Yang et al. (82)	Walking speed, temporal symmetry (stance ratio, swing ratio, swing-stance ratio, overall symmetry ratio)	13 stroke patients (with unilateral lower limb weakness; able to walk independently; and could follow instructions)	23.4 ± 15.1 months	Two IMU sensors (MicroStrain Inc., Williston, USA); shank-mounted; 10 m walking test; 3 trials; self-selected speed	Subjects' walking speed was comparable with other studies on stroke; Gait symmetry measurements were consistent with previous studies.
Nadeau et al. (40)	Spatio-temporal parameters, kinematics, kinetics	Provides a comparison with literature in terms of the actual values for healthy	n/a	Optotrak system (Northern Digital Inc., Ontario, Canada)	Kinematics:- lower limb joint motion profiles similar to those of healthy individuals, but with reduced peak amplitudes; Kinetics:- Asymmetric pattern, and reduced peak moment and powers on the affected side.
Trojaniello et al. (83)	Gait velocity, stance time, swing time, step time, stride time	10 hemiparetic subjects, 10 subjects with Parkinson's disease, 10 subjects with Huntington's disease, and 10 healthy elderly subjects	n/a	Single IMU (Opal™, APDM); lower-trunk mounted; GAITRite (12 m); single trial; self-selected, comfortable speed	Temporal parameters measured were less accurate due to the presence of missed/extra gait events; Post-stroke gait analysis using single IMU is found to be challenging.
Parisi et al. (84)	Gait cycle time, stance time, swing time, initial double support time, terminal double support duration, cadence, velocity, step length, stride length	5 hemiparetic stroke patients and 3 healthy controls	n/a	Single IMU (Shimmer, Dublin, Ireland) placed at lower trunk; optoelectronic motion capture system (ELITE 2002, BTS S.p.A., Milano, Italy) for validation; 2 force plates; 12 m hallway; 1-3 trials; self-selected speed	Low-cost system for accurate measurement of spatiotemporal features.
Wüest et al. (85)	Gait velocity, cadence, stride length, gait limb phase, gait stance phase, gait peak swing velocity, gait asymmetry	14 stroke patients (ischemic or hemorrhagic; free from musculoskeletal illness, cardiovascular disorders, or other neurologic diseases) and 25 nondisabled controls	any stage after stroke	8 body-fixed inertial sensors (Physilog, GaitUp; Lausanne, Switzerland); 2 sessions each with 3 trials; Timed Get-Up and Go task;	Excellent test-retest reliability; IMU-based timed Get-Up and Go can distinguish stroke patients from nondisabled controls.
Zhang et al. (86)	Path length, strike angle, lift of angle, maximum angular velocity, stance ratio, load ratio, foot flat ratio, push ratio	16 stroke patients (ischemic or hemorrhagic) and 9 healthy controls	5 months to 11 years (median 20 months)	Inertial sensors (MTw Awinda, Xsens Technologies B.V., Enschede, The Netherlands), shoe and lower-back mounted; 6 Minute-Walk-Test	Symmetry assessment using a single 3D accelerometer on low back shows good discriminative power compared to the one based on spatiotemporal parameters derived from two feet sensors.
Rastegarpanah et al. (87)	Step speed, step length, step time, joint angles of hip, knee, and ankle, peak ground reaction forces	4 stroke patients with hemiparesis, and 4 healthy controls (no history of neurological disorders or brain damage)	n/a	VICON MX System; Kistler force plate; 10-meter walk; 6 trials	Effect of targeting motor control on spatiotemporal parameters of gait in healthy controls as well as stroke patients; effect on peak ground reaction forces in stroke patients.
Solanki et al. (88)	Stride length, step length, stride time, step time, single support time, swing and stance phase duration, symmetry index	9 post-stroke patients and 15 healthy controls	1 to 48 months	Shoe FSR (Force Sensing Resistors), paper walkway, VICON (Vicon Motion Systems Ltd, Oxford, United Kingdom)	Design of a cost-effective and portable Shoe FSR device for gait characterization using spatiotemporal data; applicable for outdoor use.
Latorre et al. (89)	spatiotemporal and kinematic	82 post-stroke (ischemic or hemorrhagic) patients (age≥10, able to walk 10 m with/without assistance, able to understand instructions) and 355 healthy subjects (age≥10, no history of musculoskeletal or vestibular disease and/or prosthetic surgery)	748.55 ± 785.12 days	Kinect v2; at a comfortable speed	The system showed excellent reliability, validity, and variable sensitivity, thus can be used as alternative to expensive laboratory-based assessment systems, although its sensitivity to kinematic measurements is limited.
Wang et al. (90)	plantar pressure difference (PPD), step count, stride time, coefficient of variation, phase coordination index (PCI)	18 hemiparetic patients and 17 healthy adults	n/a	textile capacitive pressure sensing insole with a real-time monitoring system; 20 m long corridor;at a comfortable speed	In comparison with healthy adults, stroke patients showed higher PPD, larger step count, a larger average stride time and a lower mean plantar pressure on the paretic leg, increased plantar pressure in the toe region and lateral foot, and a threefold higher PCI. This study further confirmed the clinical applicability of textile insole sensors.
Rogers et al. (91)	peak plantar pressure and contact area	21 stroke patients (≥3 months post-stroke, able to walk 10 m independently with or without a walking aid, had no other co-existing neurological condition)	≥3 months	Tekscan HR Mat (TekScan™ South Boston, USA); 3 walking trials; self-selected comfortable speed; 2 test sessions in 2 weeks apart	Plantar pressure analysis protocol resulted in good to excellent repeatability for foot regions, except for toes.

### 4.1. Conventional Approaches to Gait Characterization

The first documented experiments on gait analysis with major contribution to muscle and tendon biomechanics date back to the seventieth century conducted by Giovanni Alfonso Borelli (1,608–1,679) the Italian physiologist, physicist, and mathematician. Richard Baker (92) provides a comprehensive review of the history of gait analysis techniques before the emergence of modern-day computers. Rudimentary experiments involving only the use of stopwatches, measuring tapes, and basic telescopes were able to make significant contributions toward the quantitative study of gait.

Gait analysis generally includes the assessment of spatiotemporal parameters, such as gait velocity, step length, stride length, single-limb support time, double-limb support time, swing duration, and cadence by direct observation or from video tapes (93). These measures, as compared and discussed by McGinley et al. (93) have varied accuracy. Dynamic data includes both kinetic and kinematic data, such as vertical ground reaction forces, plantar pressure distribution, joint reaction forces, moments and power, as well as kinematic data describing joint angular motion, and EMG data reflecting muscle activity patterns.

In addition to qualitative observational/visual gait using the naked eye and/or video images to evaluate gait performance and functional improvement (Table 2), conventional post stroke gait measurement techniques include footprint methods to measure spatial parameters and stop watches for temporal features (94, 95). Although these techniques are simple, inexpensive, and relatively reliable, the subjectivity of measurement remains a major concern, particularly in the assessment of pathologic gait.

### 4.2. Technology-Driven Approaches to Gait Characterization

In the past two decades, the field of gait assessment and analysis has witnessed a remarkable technological advancement, particularly in gait assessment technology. Instrumented walkways, despite their relatively high cost, are now widely used, both in research and to a limited extent clinical practice (20, 79, 80, 83). These systems include low-profile floor walkway systems equipped with grids of embedded sensors below the surface, which record foot-strike patterns as a function of time and space as an individual walks across the platform, and dedicated software which computes the various spatiotemporal gait measures. Patterson et al. (79, 80) used a GAITRite mat (CIR Systems Inc., New Jersey, USA) to measure spatiotemporal data toward the evaluation of post-stroke gait symmetry. Trojaniello et al. (83) employed GAITRite as a reference system to measure temporal parameters to validate the findings from inertial sensors. A similar product, Walkway™(Tekscan Inc., South Boston, USA) offers dynamic plantar pressure in addition to spatiotemporal data. The company recently launched the Strideway™, a modular gait analysis platform featuring both spatiotemporal and plantar pressure assessment platforms (88). Such instrumented mats involve less setup time and are generally simple to operate. However, they require a specific operational environment and are restricted to trials involving over-ground tasks.

Marker-based optical motion capture (Mocap) systems are well recognized to be an effective technique for obtaining 3D kinematic movement data. Passive Mocap systems [e.g., Vicon (Vicon Motion Systems Ltd, Oxford, United Kingdom) and ELITE optoelectronic system (BTS S.p.A., Milano, Italy)], include retro-reflective markers (that reflect the light emitted by high-resolution infrared cameras) attached to specific anatomic landmarks. The location of the marker is identified by decoding the camera images. Here, the markers must be calibrated for identification before the recording session commences. Active systems (e.g., Optotrak motion capture system; Northern Digital Inc., Waterloo, Canada), on the other hand, use light-emitting diode (LED) markers (reflect their own light powered by a battery), which are automatically identified. Rastegarpanah et al. (87) used a Vicon system in conjunction with a Kistler force plate as part of their experimental study on a stroke population to investigate the significance of targeting effects on gait parameters including step speed and step length. Parisi et al. (84) employed the ELITE 2002 to acquire spatiotemporal data and compared it with IMU data as part of their validation study on both hemiparetic stroke patients and healthy controls. An Optotrak-based framework was proposed by Nadeau et al. (40) to investigate the kinematic changes in persons with post-stroke gait impairment. The patients were instructed to walk over-ground after being outfitted with markers, while the kinematic data was captured and processed by the system (40). Further, in (81), the Optotrak system was proposed as an efficient tool for capturing the ground truth data associated with lower-limb joint angles in a study involving chronic stroke patients. In contrast to the over-ground trials carried out in the earlier studies, this experimental work assessed gait during a typical stair ambulation task. Unlike walk mats, motion capture systems can be used for trials involving complex tasks, where analysis of motion in multiple planes is vital. In the context of clinical relevance, although such systems yield extremely accurate reliable data, operational factors including infrastructure, non-portability, high cost, additional time required for initial set-up and calibration, operational complexity, and restrictions to indoor setup impose hurdles to their functional deployment in clinics and rehabilitation centers (88). Therefore, more portable cost-effective alternatives, such as Microsoft Kinect became the application of choice (89). This system is based on a depth sensor-based markerless motion capture solution. Less expensive 2D motion capture systems (e.g., LoCam 51 camera), as well as video analysis systems (e.g., Peak Motus Video Analysis system), were also reported in the literature with acceptable resolution (75, 76).

Optoelectronic systems (e.g., Optogait^®^, Microgate, Italy) have also been used to capture spatiotemporal gait parameters. These mainly consist of a transmitting and a receiving bar containing an infrared light. Interruptions of the communication between the emitter and receiver are detected by the system to calculate the various gait parameters. Iosa et al. (96) used an Optogait system to compute the spatiotemporal parameters, including walking speed, stride duration, stance phase, swing phase, and double support phase in a study involving patients with subacute stroke, as well as healthy subjects.

Various techniques are available to measure kinetic parameters, including ground reaction forces, and joint moments and powers. Instrumented walkways offer dynamic pressure mapping but are expensive. Force plates are also used in various gait analysis studies (81, 84, 87, 97). Chen et al. (98) developed a novel remote sensing technology called “Electrostatic Field Sensing (EFS)” for measuring human gait including stepping, walking, and running, and further extended the work to post-stroke gait. This technology is credited with several advantages, such as being non-contact, affordable, and allows long-time monitoring (99).

Shoe insole systems represent another category of gait quantification tools and techniques. These systems are designed to allow for the recording of both dynamic plantar pressure and spatiotemporal data. F-scan (Tekscan Inc., South Boston, USA) is an ultra-thin in-shoe pressure measurement system utilizing Force-Sensitive Resistive films (FSR) technology (100). Focusing on portability, cost-effectiveness, and applicability to outdoor setting, Solanki and Lahiri (88) developed FSR-based shoes (*Shoes*_*FSR*_) that offered detailed gait characterization including abnormal gait, such as observed in post-stroke. In this design, two FSR sensors were placed at the heel region spaced at 30 mm apart, and one at the toe. Maintaining the complexity of the sensing circuit to the minimum, this system successfully extracted various spatiotemporal features, including stride length and stride time by detecting gait events, such as heel strike, heel-off, toe strike, and toe-off. A recent study identified that textile capacitive pressure sensing insole (TCPSI) is a promising tool for characterizing post-stroke gait patterns (90). Several gait parameters, including plantar pressure difference (PPD), step count, stride time, coefficient of variation, and phase coordination index (PCI) were evaluated, and results were compared with normal gait. The results confirmed that textile wearable sensors may be used as a gait evaluation tool, external feedback gait training device, and a compact gait analyzer for both stroke patients and healthy subjects.

More recently, wearable sensor-based gait analysis has become popular due to its ultra-small sensor size and low cost. Miniature sensors can be attached directly to body segments, and recording can be done without the need for a sophisticated laboratory environment. These systems have potential applications in both research laboratories and clinical settings and are either based on individual sensor fusion elements (accelerometers, gyroscopes, force/pressure sensors, EMG, inclinometers, goniometers), or combined together as an integrated IMU to measure gait characteristics (99, 101–103). IMU technology successfully integrates accelerometers, gyroscopes, and magnetometers within a single unit, and can estimate the spatiotemporal and kinematic parameters from the recording of acceleration forces, angular velocity, and magnetic field data (52). The technology has also been found to be useful for the analysis of pathological gait, such as post-stroke gait. Laudanski et al. (81) measured the range of motion of the lower limb joints of individuals with post-stroke gait impairment using seven IMU sensors placed at different body segments. He suggested adopting new improved calibration techniques applicable for IMU protocol to further extend this technology to a stroke population. Trojaniello et al. (83) tested the performance of a single IMU mounted at the lower trunk to measure temporal gait parameters and found that the use of a single IMU for abnormal gait characterization is challenging, as it resulted in less accurate measurements. On the other hand, Parisi et al. (84) used a single IMU for successfully estimating both spatial and temporal gait data. Further studies have reported IMU-based gait analysis and the discriminative power of the spatiotemporal parameters obtained (82, 85, 86, 104). The pros and cons, as well as the current manufacturers of the instruments are given in Table 5.

**Table 5:** Gait analysis instruments, advantages, disadvantages, and current manufacturers.

**Instrument**	**Pros**	**Cons**	**Current manufacturers**
Pressure mat	Less setup time, easy to operate	High cost, non-portable, restricted to over-ground trials, require specific operational space	Tekscan Inc. (Walkway, F-Mat), Novel Electronics Inc. (EMED)
Pressure insole	Portable, cost-effective, does not require specific operational space, useful for indoor and outdoor setup	Low accuracy compared to pressure mat	Tekscan Inc (F-Scan), Novel Electronics Inc. (Pedar)
Motion capture	Highly accurate, useful for complex tasks involving motion in multiple planes	High cost, non-portable, additional time requirements for initial setup and calibration, special training required for operating the system, restrictions to indoor setup	Northern Digital Inc. (Optotrak), Qualisys (Arqus, Miqus), Vicon Motion Systems Ltd (VICON), BTS S.p.A. (Elite, SMART-DX)
Wearable sensors	Low cost, does not require specific operational space, useful for indoor and outdoor setup, less setup and calibration time	Special algorithms required to combine multiple sensor data	Xsens (MTw), Shimmer Sensing (Shimmer3 IMU), GaitUp SA (Physilog)

### 4.3. Gait Characterization in Clinics

The clinical application of quantitative gait assessment continues to encounter a number of barriers, including the cost of equipment, installation and infrastructure, difficulties in structured multifactorial gait assessment and interpreting a vast amount of complex gait data, low organizational support, lack of knowledge and training, and reliability and validity of the tools (105).

To overcome these limitations, researchers have proposed various low-cost gait analysis protocols with application to clinical practices (106, 107). This includes the use of commercially available technologies to develop clinically-relevant gait analysis systems with accepted levels of accuracy. On the other hand, novel wearable technology, such as inertial measurement units, are found to be cost-effective for clinical use and combined with AI-based techniques, evaluation and interpretation of the acquired gait parameters can become accessible to non-experts (103, 108). In (103), Caldas et al. presented a systematic review of gait analysis methods that use inertial sensors and adaptive algorithms. This paper has highlighted that various gait kinematic features could be acquired reliably using IMUs, but more work is needed to standardize the evaluation and report the results. Although the current research suggests promising results with regards to IMU-based gait analysis integrated with AI techniques, further assessment is needed prior to clinical implementation. As reported by Wikström et al. (108), the most widely adopted techniques include classification methods, dimensionality reduction, clustering, and expert systems. It is anticipated that these forthcoming assessment/evaluation tools, and technologies will improve clinical decision-making process and enable clinicians to devise specialized rehabilitation strategies that will be available to use in clinical settings and economical to implement. Although the value of innovation in clinical gait assessment, including post stroke, promises a paradigm shift toward data-driven precision/personalized rehabilitation, the various logistical challenges including clinical infrastructure and training, as well as the identification of target patients remain issues open for discussion.

## 5. Post-Stroke Gait Data Processing and Analysis Techniques

This section reviews both traditional and technology-driven methods of data processing and analysis protocols adopted by various researchers in the context of gait analysis. Technology driven approaches into gait characterization provide a vast amount of data that cannot be simply interpreted in clinical practice. Therefore, in addition to the measurement technology, we review data-mining technology, that allows vast amount of data to be interpreted in a meaningful time-frame for the use by the clinicians during the rehabilitation.

### 5.1. Conventional Approaches to Gait Data Processing and Analysis

Numerous computational techniques, including traditional statistical tests have been applied to analyzing quantitative gait data obtained from instrumented gait recording technology (79, 80, 109–115). For example, Lin et al. (110) employed stepwise regression analysis to identify important impairments in persons with stroke (muscle strength of plantarflexors and dorsiflexors, spasticity index, passive stiffness of plantarflexors, and position error) associated with gait parameters, and Pearson correlation coefficients to study the relationship between the gait parameters (i.e., gait velocity and spatiotemporal symmetry) and impairment parameters. Cruz et al. (113) based their analyses on multiple linear regression models. ANOVA tests were used to determine the variability between different stages of post-stroke, as well as the significant effect between variables within the same group (80). Another study used an unpaired *t*-test to compare stroke patients with controls, and further adopted correlation tests to examine the relationship between different symmetry measures, including symmetry ratio and symmetry index (79). Further, stepwise linear regression, as well as descriptive statistics (mean and standard deviation) and correlation tests were adopted to study the behavior of the hip and ankle joints during the swing phase of the gait cycle in individuals with post-stroke gait impairment (116). In (83), the difference between mean absolute values of initial contact timings associated with different gait event detection methods was calculated using the Wilcoxon signed-rank test. This method was further applied to examine the difference between the gait characteristics for affected and unaffected limbs. In addition, the Wilcoxon rank-sum and Friedman tests were also applied in the same work. Although such methods involve less computational cost, they often fail to incorporate a variety of high-dimensional inter-related data obtained from different devices requiring more complex statistical analysis methodology (108).

### 5.2. Technology-Driven Approaches to Gait Data Processing and Analysis

The field of big data provides powerful techniques to systematically analyze and extract information from instrumented gait data that are too large, heterogeneous, and/or complex to deal with traditional statistical approaches. Machine learning techniques, including predictive analytics and data mining techniques, have been incorporated to characterize both normal and pathological gait. Predictive analytics uses data modeling to understand trends in data to predict future outcomes (e.g., SVM). Data mining approach, on the other hand, helps to discover new patterns in data (e.g., clustering) (117).

Principal Components Analysis (PCA)-based techniques have widely been used as part of gait data analysis. Olney et al. (75) implemented PCA for dimensionality reduction, (118) applied the same technique on the kinematic data obtained from 27 stroke patients, and (119) employed Functional PCA (FPCA) to understand the relationship between velocity and functional assessment scales.

Several attempts have been made to classify the patterns of hemiplegic gait, particularly toward planning dedicated rehabilitation strategies. Most recently, the applicability of artificial intelligence has been explored. The most common adopted machine learning classifiers include Support Vector Machines (SVM) and Artificial Neural Networks (ANN). Lau et al. (44) implemented SVM, ANN, and Radial Basis Function neural networks (RBF) classifiers to classify five different walking conditions (level ground, stair ascent, stair descent, upslope, and downslope) for hemiparetic patients. The SVM based classifier outperformed ANN and RBF methods with a highest overall classification accuracy of 97.5%. Kaczmarczyk et al. (120) applied ANN for classifying post-stroke gait patterns.

Clustering analysis has also been used to classify the gait patterns of stroke patients by employing spatiotemporal and kinematic parameters as input features. The gait velocity, peak knee extension during mid stance, and peak dorsiflexion during swing phases were identified as key features that best discriminated the groups (121). A similar approach was used by (122) for post-stroke gait classification. Another study proposed a feature extraction algorithm for plantar pressure images obtained from stroke population (123).

## 6. Post-Stroke Gait Parameters

This section will review various gait and physiological parameters used to characterize post-stroke gait.

### 6.1. Spatiotemporal Parameters

Spatial and temporal parameters of post-stroke gait are significantly different from those of healthy individuals. Von Schroeder et al. in 1995 conducted a study on 49 ambulatory stroke patients and 24 controls to evaluate the changes in the spatiotemporal parameters and motion patterns (74). The patients who had no hemiplegia and those with bilateral symptoms were analyzed separately. The controls included in the study had either transient ischemic episodes or asymptomatic carotid stenosis but maintained symmetrical gait without walking support. Both comprehensive (gait velocity, cadence, stride length, gait cycle, total double support duration) and unilateral (single support duration, duration of swing phase, duration of stance phase) parameters of gait were analyzed. Compared to age-matched controls and non- disabled individuals, post-stroke patients exhibited decreased walking speed and cadence, as well as increased gait cycle and double limb support. It was also observed that the hemiplegic limb of patients spent more time in swing and stance, while their unaffected limb spent more time in stance and single support as compared to the controls. In addition, less time was spent in stance and more time in the swing phase on the paretic limb as compared to the contralateral side. However, the general gait parameters were observed to improve over time, with the exception of the asymmetrical patterns which did not change. Table 4 reports on various gait parameters applicable to post-stroke gait analysis as well as their significance.

In an attempt to recommend the most suitable gait measures for standardization, Patterson et al. (79) compared different asymmetry measures that describe post-stroke gait, including the symmetry ratio, symmetry index, log transformation of the symmetry ratio, and symmetry angle. The study recruited 161 unilateral stroke patients and 81 healthy controls. They found that no particular individual measure distinguished stroke patients better than others. However, they suggested symmetry ratio as a suitable measure based on its clinical utility. The most useful parameters of gait included step length, swing time, and stance time. Measures of gait speed and symmetry across the post-stroke stages also reflected both spatial and temporal symmetry deterioration in the later phases of post-stroke, whereas velocity, neurological impairment, and motor deficit did not change (80). These findings are in line with (124) but contradict (74), who found an improvement of gait velocity and cadence, especially during the first 12 months following a stroke, with no changes observed in asymmetry patterns. These contradictory findings may be due to selection bias and sample size variability that can affect the results in such studies. Although not explicitly reported in these papers, compensation strategies may have played a role in improving speed without enhancing symmetry.

A study on gait velocity and temporal symmetry (temporal stance ratio, temporal swing ratio, temporal swing-stance ratio, overall temporal symmetry ratio) involving 13 stroke patients with unilateral lower-limb weakness reported reduced gait velocity during a standardized 10-meter walk test as well as deterioration in temporal asymmetry, comparable with other related studies on individuals with post-stroke gait impairment (82). It has been reported that difference in spatiotemporal parameters could be influenced by gait speed, suggesting speed-matched trials for better post-stroke gait characterization. Consistent differences in the spatiotemporal parameters were noticed between stroke patients and healthy controls at speed-matched assessment (125, 126).

In summary, the literature agrees that the spatiotemporal characteristics of post-stroke gait include reduced step or stride length, increased step length on the hemiparetic side, slightly wider base of support, slightly greater toe-out angle, reduced walking speed and cadence. Stride time, stance period on both lower limb, and double support time are increased. In addition, less time in stance and more time in swing phase for the paretic side, as well as asymmetries in spatial and temporal factors have been reported (77).

Silver et al. assessed the improvement in the functional aspects of gait in patients with chronic hemiparesis after undergoing task-oriented aerobic training (76). The temporal parameters, including walking speed, cadence, gait symmetry (intralimb stance-swing ratio, interlimb stance duration ratio, interlimb swing ratio, overall stance-swing ratio) were examined before and after training using a modified TUG task. The post-ischemic stroke patients in this study exhibited mild to moderate gait asymmetry. A significant improvement in the walking speed and cadence, as well as a reduction in the overall time required to complete the TUG task were evident with time. However, improvements in gait asymmetry and temporal sequencing remained not significant. On the other hand, the gait patterns of chronic hemiparetic stroke patients were compared between over ground and treadmill walking by analyzing relative stance time, relative single-limb stance time, stance-swing ratio, peak force, and impulse (127). It has been observed that self-supported treadmill walking improved gait symmetry in hemiparetic stroke patients.

### 6.2. Kinematic Parameters

Available evidence indicates that changes in the stance phase of hemiplegic gait are attributed to reduced mean peak extension of the hip joint in late stance, alterations in the lateral displacement of the pelvis and flexion of the knee, and decreased plantarflexion of the ankle at toe-off (72, 73). Further, hemiplegic culprits include a significant decrease in peak hip and knee flexion during the swing phase, reduced knee extension prior to initial contact, as well as decreased ankle dorsiflexion during swing. In general, the literature suggests that the joint motion profiles of hemiplegic patients are influenced by abnormal muscle activation patterns, muscle shortening (72, 73), and/or reduced walking speed (128). In some patients, it was noted that compensatory walking mechanisms may also lead to abnormal angular motion patterns, for example, knee hyperextension to compensate for stable weight support during forward propulsion (129).

### 6.3. Kinetic Parameters

Kinetic analysis of gait includes accounting for the ground reaction forces (GRF) and joint moments and powers. GRF refers to the forces exerted by the body on to the ground and are usually measured by force sensors embedded in a walking platform. Moments and powers are obtained via inverse dynamics by combining GRF measurements with kinematic data.

Post-stroke GRF patterns differ from those of healthy subjects exhibiting an asymmetric pattern, as well as decreased amplitudes of the joint moments and joint powers at the hip, knee, and ankle joints on the paretic side (40). Overall, three main types of vertical GRF patterns have been observed in individuals with post-stroke gait impairment (77, 130): (1) a force curve with two vertical peaks occurring at loading and push-off and an intermediate trough at mid-stance phase, similar to the one seen in healthy groups, (2) a relatively constant vertical force component during stance, and several irregular peaks, (3) a single vertical peak force during early stance, which gradually reduces to zero during the late stance phase. In addition, a high correlation between the GRF pattern and the foot contact pattern (heel-, flatfoot-, forefoot-initial contact) of stroke patients has also been observed (131). These kinetic patterns can be easily computed from wearable sensors as detailed in (132).

It has been consistently reported that in stroke patients, the net positive moment and power at the hip, knee, and ankle joints follow profiles that are similar to those of healthy individuals while walking at self-selected comfortable speed and low gait speed, although with reduced amplitude in both limbs, and smaller amplitude on the affected side as compared to the non-affected side (40). Moreover, the amplitude of several kinetic parameters (e.g., hip power bursts) was found positively scaled to gait velocity or other functional ability (e.g., plantarflexors strength) measures of stroke patients (65). Some studies have revealed the applicability of plantar pressure dynamics in understanding foot and ankle movement patterns (65, 133–135). Spatial and temporal distribution of foot plantar pressure and the displacement of the Center of Pressure (CoP) are significant findings in this context. Several abnormalities, including lower plantar pressure peaks for the paretic foot as compared to the non-paretic foot (133), early paretic forefoot contact (134), asymmetries in the spatial and temporal distribution of plantar pressure along the plantar surface of the foot between affected and unaffected limbs, as well as the variability of the CoP, have been reported in individuals with post-stroke gait impairment (65, 134, 135). A recent study (91) addressed the repeatability of plantar pressure assessment in stroke patients and claimed good to excellent repeatability for the foot regions with exception to toes.

### 6.4. Electromyographic (EMG) Parameters

Surface EMG is a noninvasive technique used to capture muscle activity, which could provide further insight into post-stroke gait abnormalities since during walking EMG data reveals characteristic patterns of neural activation associated with each involved muscle in terms of onset timings, burst durations, and levels of activations (136). However, EMG-based assessment for gait asymmetry is not well explored as compared to assessment using other typical gait attributes. Similar to other gait parameters discussed above, EMG signals from stroke patients reveal abnormal amplitude and timing as compared to healthy controls. Flexor and extensor mechanisms seem to contribute to such abnormal behavior (137). Hesse (78) reported the most common EMG abnormalities including high tonic activity of the tibialis anterior, early onset of the gastrocnemius, delayed onset of the vastus lateralis, and highly paretic gluteus medius muscle activity at gait onset.

### 6.5. Additional Parameters

Autonomic dysfunction is another common complication after stroke, which inspired investigations regarding the role of Heart Rate Variability (HRV) (138, 139). Korpelainen et al. showed that HRV changes were present in patients with hemispheric and medullary unilateral brainstem stroke (138). Similarly, recovery of parasympathetic function was observed from the third day after the onset of stroke, while both parasympathetic and sympathetic functions recovered by the seventh day, depending on the extent and laterality (midline versus lateral) of stroke. It is therefore recommended that autonomic function be taken into account during the first week after stroke to prevent cardiac complications, such as postural hypotension during physical therapy (139). Another study explored the significance of HRV in predicting post stroke motor recovery and revealed a strong positive correlation between HRV and the movement of the affected extremities (140). Short-term and long-term variability of heart rate analysis of post-stroke patients also reported changes in HRV associated with impaired renal function which correlated with stroke severity.

Among other neurocontrol parameters, toe clearance (109, 141), foot clearance (142), claw toes (143), and mechanical energy cost (65), in association with post-stroke gait characteristics, were also reported to be useful in improving treatment outcomes and monitoring post-stroke rehabilitation. Particularly, Begg et al. (141) provided evidence that biofeedback training based on minimum toe clearance was useful in reducing tripping risk of post-stroke patients.

Analysis of additional biomechanical parameters suggested that multi-joint abnormalities (e.g., reduced flexion at knee and ankle) (142) contributed to the lower foot clearance in post-stroke individuals. In (143), 46% of 39 hemiplegic patients exhibited claw toe mostly before the end of the third month post-stroke. Claw toe was significantly correlated with equinus and/or varus foot. In addition, the mechanical energy cost was found be higher in post-stroke patients as compared to healthy subjects at similar walking speed (65).

## 7. Gait Analysis and Artificial Intelligence

Expert Systems (ES) represent early applications of analytical tools in Artificial Intelligence (AI). They consist of a combination of a database with a knowledge base, as well as rules including logic operators that utilize probability theory to provide possible outcomes as part of a user interface (144, 145). Expert systems initially require users to define variables and appropriate diagnostic values, as well as associated rules, confidence factors, and user specified questions. The expert system then generates a set of questions that guide the user toward a systematic diagnosis or evaluation (146). An important feature of ES is that functional evaluation, which is based on clinical observations, can be standardized by incorporating a set process. ES have been applied in numerous disciplines including general biomechanics, sports biome- chanics, orthopedics, and gait analysis for improving movement techniques, diagnostics, rehabilitation, and treatment. By far the most common use is in gait analysis (147). Early studies included automated detection of gait events using inductive learning (148) and an integrated gait analysis framework (149, 150). Further advances in the use of ES came with the advent of multi-sensor technology and analytics including video capture of movement with reflective sensors placed on the body, force plate derived features and physiological/neuromuscular features (151). Use of more ANN-based classification superseded ES with AI demonstrating better accuracy as compared to statistical expert systems models. ANN consists of a series of interconnected nodes that can be either designed as a single layer or multilayer approximating the relationships, or adaptive weightings determined from a training set, between input and output measures, which can then be applied to unseen data (150). Lugade and colleagues applied self-organizing maps (SOM) or so-called Kohonen maps to estimate gait balance control in the elderly using clinical evaluations (152). ANNs also include feedforward or feedbackward backpropagation neural networks (153). Further applications in gait included estimating joint kinetics and kinematics using electromyography and determining spatiotemporal gait patterns from EMG recordings to determine falls risk or control measures for retaining balance during gait (154–156).

As the volume of data from the variety of nowadays readily available body sensors used to quantify human gait and movement, including electroencephalography, electro-oculography, electro-cardiography, and electromyography video and force plate data, substantially increases, more sophisticated modeling is needed to quantify and interpret complex network physiology (157). In addition, today's advanced use of computer science has established novel features describing gait movement associated biomechanics, moving from discrete data to more realistic dynamic representations (158, 159). Multivariate statistical analysis, machine learning methods including Support Vector Machines (SVM) have been recently extended to Deep Artificial Neural Networks such as Layer-wise Relevance Propagation (LRP) methods to provide numerical data on the contributions of variables included in the model (160).

There remains a gap in literature regarding advanced neural networks that deal with gait individualized pattern characteristics. Since those are unique for each individual, they require models that not only include conventional features based on training static features of a sample population, such as maximum joint angles, but also the inclusion of dynamic variables such as the change of joint angle over time in conjunction with sophisticated data reduction and individualized feature selection of the most relevant gait characteristics in the context of personalized medicine (161). Applying new statistical learning algorithms, including deep learning combined with large data sets, has led to the emergence of Explainable Artificial Intelligence (XAI) designed to gain information about the individual features included in the final AI classification. However, this approach has had limited applications with clinicians who required decision support algorithms to provide information on causality as well (162). Table 6 provides an overview of the up-to-date AI techniques used in various literature resources.

**Table 6:** An overview of the AI techniques applicable for gait analysis.

**References**	**Input parameters**	**Technique**
Lau et al. (163)	Kinematic data	Support vector machines (SVM), Artificial neural network (ANN), Radial Basis Function network (RBF), and Bayesian Belief Network (BBN).
Lai et al. (56)	Spatiotemporal, kinematic, kinetic, and EMG data	Signal processing and computational intelligence methods.
Lau et al. (44)	Kinematics data	SVM, ANN, RBF.
Kaptein et al. (164)	kinematic and physiological data	Analysis of variance (ANOVA) supplemented by logistic and partial least squares (PLS) regressions.
Laroche et al. (165)	Kinematic trajectories	SVM.
Karg et al. (166)	time series gait data	Hidden Markov Model (HMM).
Cippitelli et al. (167)	body joint trajectories	Algorithm based on anthropometric models.
Joyseeree et al. (168)	Spatiotemporal data	Random Forest (RF), boosting, Multilayer Perceptron (MLP), and SVM.
LeMoyne et al. (169)	Temporal and kinetic data	SVM.
Ferber et al. (170)	n/a	n/a.
Osis et al. (171)	Kinematic data and ground reaction forces	Principal Component Analysis (PCA).
Zeng et al. (172)	Vertical GRF	RBF networks.
Hannink et al. (173)	Spatiotemporal data	Deep convolutional neural networks.
Caldas et al. (103)	IMU data	artificial intelligence (AI) algorithms [e.g., artificial neural networks (ANN) and hidden Markov models (HMM)].
Park et al. (174)	Spatiotemporal and plantar pressure	Random forest classification.
Pham and Yan (175)	Vertical GRF	Tensor decomposition.
Ertelt et al. (176)	GRF	Bayesian regulated neural networks.
Haji Ghassemi et al. (177)	Inertial data	Peak detection, two variants of dynamic time warping (DTW) methods [Euclidean DTW (eDTW) and probabilistic DTW (pDTW)], and hierarchical hidden Markov models (hHMM).
Zhan et al. (178)	Stride length	A rank-based machine-learning algorithm called disease severity score learning (DSSL).
Zhang et al. (179)	GRF	SVM.
Bastien et al. (180)	Ground reaction forces (GRF)	A predictive linear model of the fore-aft GRF.
Galbusera et al. (181)	review article	Machine learning and deep learning.
Jiang et al. (182)	Inertial data, GRF	Random forest learning.
Nguyen et al. (183)	Inertial data	PCA, SVM, ANN.
Prado et al. (184)	Temporal data	Recurrent Neural Network classifier model.
Waugh et al. (185)	Accelerometer data	Canonical dynamical system (CDS)Fourier series.
Jauhiainen et al. (186)	Kinematic data	Cluster analysis.

## 8. Limitations

This review aimed to summarize available published work on the assessment, quantification, as well as analysis of gait dysfunction associated with post-stroke gait. The focus was to highlight recent technology-driven gait characterization and analysis approaches and their applicability to clinical practice toward data-driven informed treatment and precision rehabilitation strategies. As such, this article may have not covered the complete biomedical aspects of stroke assessment and/or rehabilitation. A non-systematic search methodology was selected in order to broaden the scope and integration of the three aspects of focus (assessment, quantification, and analysis). In addition, we do not recommend any specific protocol over the other, as most of the papers incorporate different inclusion/exclusion criteria for subject selection, as well as different sampling sizes, which renders comparisons unrealistic.

## 9. Conclusion and Future Work

The main contribution of this work is providing a multidisciplinary comprehensive review on post stroke gait assessment and analysis toward bridging the gap between research gait studies and clinical applications. The main goal is to offer a practical resource on the multidimensional aspects of post stroke gait focusing on novel tools and technologies for quantitative assessment that can be feasibly incorporated into clinical practice.

As generally agreed upon by many clinicians, quantitative gait analysis outperforms traditional observational scales as it generates unbiased outcomes that can be used as benchmarks for rehabilitation. Instrumented gait analysis has wide potential applications throughout the different phases post-stroke. For example, as a diagnostic tool to quantitatively assess the severity of a stroke and establish measurable benchmark parameters toward devising patient-specific rehabilitation strategies. It can also be used as means for continuously evaluating recovery while ensuring patient safety and leveraging the plasticity of the brain constrained by time during the various stroke phases. A variety of gait parameters, including spatiotemporal features, gait kinematics, kinetics, EMG patterns, toe and foot clearance, claw toes, mechanical energy cost, and HRV are available for consideration, where each signifies different aspects of motor and functional deficits associated with stroke that can be analyzed using a diverse menu of available statistical and analytical tools, including XAI. As can be observed from the literature, spatiotemporal symmetry and gait velocity are considered to be key parameters for post-stroke gait analysis. On the other hand, in recent sensor fusion approaches, multiple parameters are simultaneously assessed to reflect and evaluate the inherent complexity and variability of gait, including various physiological phenomena, such as EMG, ECG and EEG, in addition to gait. Future work is needed to recommend the best set of parameters for post-stroke gait assessment. This review also summarizes the variety of current measurement devices and tools available for acquiring gait data. The latest trend is toward smart wearable technology, which promises a paradigm shift in clinical gait assessment, including stroke, and the creation of low cost, portable, gait labs that can be transferred to the clinic for accurate and reliable dynamic gait assessment. Data analyses techniques help to understand the underlying information associated with these parameters. In a clinical context, it would be invaluable if such algorithms can predict post-stroke recovery status and time, although this still seems to be a highly challenging task. AI/NN models that include static and dynamic features, combined with sophisticated data reduction and individualized feature selection of the most relevant gait characteristics are needed to close the loop for this paradigm shift in alignment with personalized post stroke assessment and rehabilitation.

Researchers and clinicians may consider looking into the following key points:

1. Quantitative gait analysis is considered beneficial over the conventional techniques during various stages of post-stroke gait quantification, assessment, and analysis.
2. A combination of gait and physiological markers should be employed while analyzing pathological gait, such as post-stroke gait due to the complexity and variability of gait patterns involved.
3. Data mining, non-linear and AI techniques could be suitable options for post-stroke gait data analysis.

The authors are currently working on devising methods for identifying the most significant gait parameters for clinical use, cost and time-effective protocols for measuring the parameters in clinical settings, and the most effective techniques for data mining and analyses with the support of data from stroke patients. The relevant questions of the need for innovation in clinical post stroke gait assessment and identification of target patients remain open for debate. Further clinical applications of the above reviewed tools and technologies are needed to demonstrate the overall efficacy of this paradigm shift in comparison to conventional assessment methodologies.

## Author Contributions

DMM and KK conceived the idea. DMM, KK, AHK, and HFJ formulated the objective for this review. DMM designed the search strategy, conducted abstract screening and full text review, extracted the data, and drafted the manuscript. KK, HFJ, and AHK contributed to writing the manuscript. SIIIA performed a part of the literature survey, including abstract screening, full text review, and data extraction. AHK, SAW, HFJ, and KK provided significant guidance on the content of the manuscript, overall supervision, and critical feedback. All authors contributed to the manuscript revision and approved the final version of the manuscript.

## Conflict of Interest

The authors declare that the research was conducted in the absence of any commercial or financial relationships that could be construed as a potential conflict of interest.
